# Coverage of cancer services in Australia and providers’ views on service gaps: findings from a national cross-sectional survey

**DOI:** 10.1186/s12885-019-5649-6

**Published:** 2019-06-11

**Authors:** Jennifer Hunter, Caroline Smith, Geoff P. Delaney, Kate Templeman, Suzanne Grant, Jane M. Ussher

**Affiliations:** 1 NICM Health Research Institute, Western Sydney University, Campbelltown Campus, Locked Bag 1797, Penrith, NSW 2751 Australia; 2 Menzies Centre for Health Policy, School of Public Health, Faculty of Medicine and Health, The University of Sydney, Sydney, NSW Australia; 3 South-Western Sydney Clinical School, Faculty of Medicine, University of New South Wales, Sydney, NSW Australia; 4 Cancer Services, South Western Sydney Local Health District, Sydney, NSW Australia; 5 Ingham Institute of Applied Medical Research, Liverpool, NSW Australia; 6 Translational Health Research Institute, School of Medicine, Western Sydney University, Sydney, NSW Australia

**Keywords:** Oncology service, Survivorship, Health services financing, Health care survey

## Abstract

**Background:**

In response to the increasing cancer prevalence and the evolving health service landscape across the public and private health sectors in Australia, this study aimed to map cancer services and identify factors associated with service provision and important service gaps.

**Methods:**

A prospective, cross-sectional survey was conducted throughout 2016. Extensive search strategies identified Government or privately-owned, hospital or community-based healthcare organisations with dedicated cancer services. One nominated staff member from each organisation answered a purpose specific online/paper questionnaire. Descriptive statistics, standardised rates, and single level and multilevel multinomial logistic regression were used to analyse the data. Analysis was augmented with a qualitative descriptive analysis of open-ended questions.

**Results:**

From the 295 eligible organisations with a cancer service in Australia, 93.2% participated in the survey. After adjusting for remoteness, for-profit companies were significantly more likely than Government operated services to provide only one or two types of cancer services (e.g. radiotherapy) in a limited range of settings (e.g. day hospital with no in-patient or home care) (*p* < 0.001) and less likely to provide comprehensive cancer services (p < 0.001). After adjusting for ownership and the respondent’s role in the organisation, respondents located in remote regions of Australia were more likely to identify cancer services that are dependent upon specialist medical practitioners as the most important service gaps in their region (*p* = 0.003). Despite 76.0% of organisations across Australia offering some type of supportive care or survivorship services, providers identified this group of services as the most pressing service gaps in major cities, rural and remote regions alike (standardised rate: 47.9% (95%CI: 43.6–57.4%); *p* < .000). This included the need for improved integration, outreach and affordability.

**Conclusions:**

The broad range of cancer services, settings and ownership identified by this survey highlights the complexity of the Australian healthcare system that cancer survivors must navigate and the challenges of providing comprehensive cancer care particularly in rural and remote regions. Whilst the significant role of supportive care and survivorship services are increasingly being recognised, the findings from this survey support calls for innovative service models and funding mechanisms that expand the focus from preventing and treating cancer to supporting cancer survivors throughout the cancer continuum and promoting the delivery of integrated and equitable cancer care across the public and private sectors.

**Electronic supplementary material:**

The online version of this article (10.1186/s12885-019-5649-6) contains supplementary material, which is available to authorized users.

## Background

Cancer is a leading cause of mortality and morbidity in Australia, accounting for around one third of deaths and 19% of the total burden of disease [1]. For years lived in less than full health, 2.3% of the non-fatal burden is attributed to cancer. Between 2003 and 2011, the fatal burden and non-fatal burden increased by 7.5 and 28.8% respectively [2]. Over the past 30 years, all-cause cancer incidence in Australia has increased by 27% [1]. According to the CONCORD-3 Global surveillance of trends in cancer survival 2000–14, Australia has some of the highest cancer survival rates in the world [3]. Coupled with an aging population, cancer prevalence in Australia is continuing to rise, placing increasing pressure on the health and social services to provide care throughout the cancer continuum. Added to this, along with the sequelae of cancer and cancer treatment, cancer survival is associated with an increased risk of other chronic diseases and general functional decline [4–7].

The bio-psycho-social needs of people who have been diagnosed with cancer (hereafter referred to as cancer survivors), extend beyond just ‘surviving’ cancer [8]. In Australia, and for the purpose of this paper, the term survivorship care and associated services is used broadly and refers to cancer surveillance and prevention, the management of the sequalae of cancer and its treatment, and the integration of cancer care between service providers [9]. Supportive care services that are mostly provided by allied health practitioners (e.g. aboriginal health workers, complementary medicine practitioners, dietitians, exercise physiologists, occupational therapists, physiotherapists, podiatrists, psychologists and social workers) are an important component of survivorship care.

Australia has a mixed public-private health service with a large primary care workforce. The country has a national health insurance scheme that funds a baseline of primary and secondary care services. This includes a national purchasing and subsiding scheme that uses a health-economic perspective to contain the costs of selected pharmaceuticals. The public health sector is charged with the responsibility of coordinating cancer prevention, screening programs and the national cancer registry and ensuring comprehensive service provision for all cancer survivors residing in Australia. Coordination of services is mostly at the state level (of which there are seven) and regional levels such as the Primary Health Network (PHN) (of which there are 31). Increasingly, the public and private sectors are collaborating to improve the coverage of cancer service provision across Australia. Whilst the national health insurance covers the cost of all inpatient and outpatient services accessed in Government owned hospitals, the costs of accessing pharmaceuticals and healthcare services in the community are only partially subsidised. Optional private health insurance is used by some patients to further subside some of the costs of private healthcare accessed in hospital settings and some ancillary services, including allied health and other supportive care accessed in community settings.

The mixed public-private system in Australia is not without its concerns as there is potential for ‘cream skimming’ where the private sector selectively provides high profit services such as radiotherapy, chemotherapy and surgery, and transfers complex patients back into the public sector [10]. Further, there is evidence of disproportionately higher out-of-pocket costs for cancer survivors accessing private healthcare in Australia that does not necessarily correlate with higher quality care [11].

Despite the evolving health service landscape, few national studies have been undertaken that map the evolving landscape of cancer services in Australia. Of those studies conducted, the focus has been on service provision in specific geographical areas or clinical fields [12–15]. One of the most detailed studies was a 2005 survey of 161 regional and remote hospitals in Australia that administered chemotherapy [12]. Substantial service gaps were identified with only 21% of hospitals providing an inpatient medical oncology service, 7% radiation oncology, 6% surgical oncology and 24% access to an onsite palliative care specialist. Whilst most of these cancer services (90%) provided allied health and supportive care services, access was reported to be limited due to long waiting times, few or no outpatient services, high out-of-pocket costs and inadequate transport services for patients and their caregivers. More recently (in 2015), a national survey mapped supportive cancer care referral pathways and service provision in 124 hospitals with cancer services [15]. Only 28% provided either a ‘cancer-specific supportive care service’ or direct access to these services via an affiliated cancer centre. Around half (53%), had no established referral pathway and 19% referred cancer survivors (possibly on an ad-hoc basis) to external organisations or allied health practitioners.

In light of these studies, a national survey of healthcare organisations in Australia that provide specialised cancer services was conducted in 2016. The aim was to identify all hospital and community based organisations across Australia, map cancer service provision, explore the relationship of service provision with ownership and geographical remoteness of the organisation, and identify important regional service gaps from the perspective of providers.

## Methods

### Study design, sample and participants

A mixed-method, prospective, cross-sectional survey was conducted between 1 May to 15 December 2016. An extensive search strategy was employed beginning in November 2015 that aimed to identify all healthcare organisations with a dedicated cancer service operating in either the public health sector or private health sector; the latter comprising of for-profit and not-for-profit companies. Included in the sample were adult, adolescent and children services, inpatient or outpatient hospital, day-hospital, or community-based organisations. Hospices and palliative care services that were not part of a larger cancer service were excluded; as were small businesses that ran clinics or consultation rooms for healthcare professionals (e.g. oncologist’s private consultation rooms) and services that only offered support groups, counselling or information for cancer survivors.

Eligible organisations were identified from the Australian Institute of Health Welfare Australian My Hospitals database [16]; Hospitals and Aged Care Database [17]; and Australian Health Directory [18]. Additional services and sites were identified through conversations with industry experts from national cancer organisations and from survey participants. The search for community-based organisations was further augmented with a systematic, location-based Internet search using Google and Bing search engines. The search was conducted by volunteers from each state or territory who were familiar with the cancer services in their state.

Ethics approval was obtained from the appropriate university, state, hospital, and local health district committees. Having first agreed to participate in the survey, each organisation nominated a suitable staff member to answer the survey. Their contact details were provided to the research team and informed written consent was obtained.

### Data collection and questionnaire

A purpose-specific, self-administered, confidential questionnaire was designed (Additional file 1) that was pilot tested locally. The first part of the questionnaire collected information about the oncology service, such as geographical location, ownership, setting, and types of cancer services provided, and important gaps in cancer services in their region. Information about capacity such as the number of beds or patients treated was not collected due to concerns about responder burden and likely inaccuracies. The second part of the questionnaire asked questions about complementary medicine services and policies. These results will be reported elsewhere.

Participants were sent a pdf version of the survey, and a link to the on-line survey that was administered via Survey Monkey®. A follow-up reminder email was sent to non-responders every 3 weeks leading up to the final 2 weeks prior to closing the survey. Remaining non-responders were also recontacted in the final weeks of the survey.

### Data analysis

Descriptive and inferential quantitative analyses were undertaken using SPSS® Versions 24. All questions bar those inquiring about service gaps were compulsory. Missing data were excluded in the regression analysis of service gaps. Chi squared and Fisher-Freeman-Halton tests, and binomial and multinomial logistic regressions were used. Postcode location was used to code the data according the Australian Bureau of Statistics Postcode 2012 to Remoteness Area 2011 [19], and the 31 national Primary Health Network (PHN) regions. PHN standardised rates of unmet need were calculated to adjust for uneven numbers of responses for each region and a hierarchical logistic regression was used to adjust for PHN cluster effects when calculating odds ratios. ArcGis 10.00 software was used to generate the geographical map to display the number of organisations per Australia Post Code [20].

The open-ended questions and comments about service gaps and unmet needs were exploratory as this was the first time such questions had been asked of providers. Coding was descriptive. The results were independently coded by authors CS (an academic researcher with an allied health background) and JH (a primary care physician and public health/health services researcher who has clinical experience working in multidisciplinary teams). Any discrepancies were resolved through further discussion with the research team that included an oncologist. Data was entered into spreadsheets, compared and then jointly coded to into categories. Descriptive quantitative and qualitative methods (i.e. mixed-method) were used to summarise and present the results.

## Results

### Survey response

A total of 366 healthcare organisations were shortlisted, from which 295 healthcare organisations met the inclusion criteria and were confirmed to have a dedicated cancer service and invited to participate in the survey. The overall response rate from the organisations was 93.2% (*n* = 275). All of the 275 participants who were nominated to answer the questionnaire on behalf of their organisation completed the questionnaire. Response rates differed by state and territory (hereafter, all referred to as states) (Fisher-Freeman-Halton Test *p* = 0.02) with the lowest response rate in the Northern Territory (66.7%) and the highest in Tasmania (100%). Response rates were significantly lower in rural regions (88.5%), followed by organisations located in major cities (92.3%) and highest in remote regions (98.9%) (Fisher-Freeman-Halton Test *p* = 0.03). No significant differences were observed in response rates according to the ownership of the organisation nor the cancer service setting.

### Participant characteristics

Just over half of the 275 individual respondents (55.6%, *n* = 153) reported a dual role in the organisation as both a healthcare professional (HCP) and administrator/manager, 73 (26.5%) worked as a HCP only, and the remaining 49 (17.8%) worked in administration/management only.

### Location, ownership, services provided and settings

**Fig. 1:**
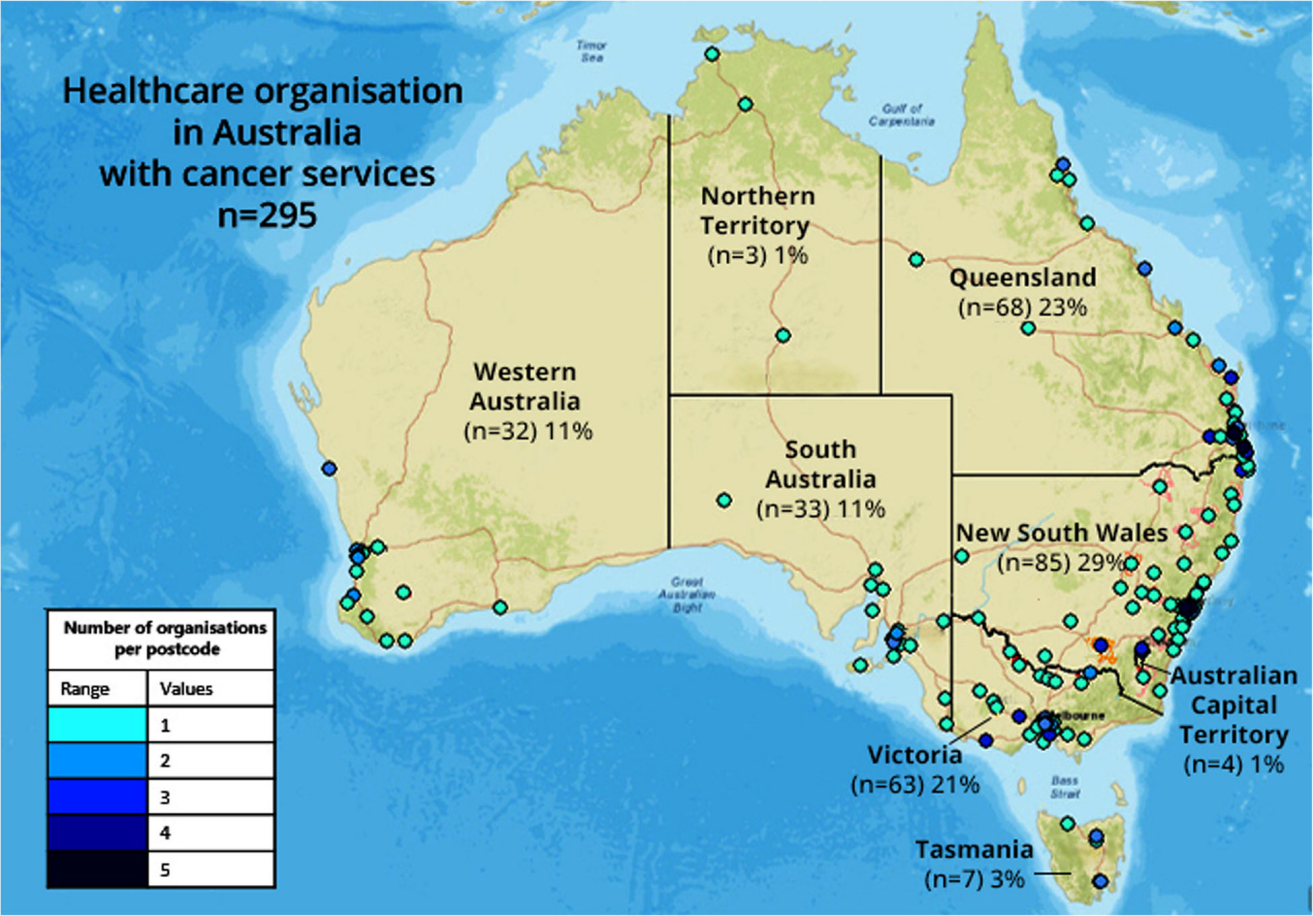
Location and density of cancer services in Australia. Created in ArcGIS [20] using open-source Esri map

Cancer services were located in the most populous states and regions (Fig. 1). Half of the participating healthcare organisations (49.5%) were public, government operated services and 28.4% were owned by a for-profit company and 22.2% by a not-for-profit company (Table 1).

**Table 1:** Location, ownership, service settings and response rates of Australian cancer services

Healthcare Organisations with cancer services	All eligible services		Responders Response rate	
	n	%	n	%
State/Territory* (*p* = 0.02)				
Australian Capital Territory	4	1.4 %	3	75.0 %
New South Wales	85	28.8 %	82	96.5 %
Northern Territory	3	1.0 %	2	66.7 %
Queensland	68	23.1 %	67	98.5 %
South Australia	33	11.2 %	28	84.8 %
Tasmania	7	2.4 %	7	100 %
Western Australia	32	10.8 %	28	87.5 %
Victoria	63	21.4 %	58	92.1 %
Remoteness* (*p* = 0.03)				
Major cities	117	39.7 %	108	92.3 %
Rural	87	29.5 %	77	88.5 %
Remote	91	30.8 %	90	98.9 %
Ownership				
Government operated	148	50.2 %	136	91.9 %
For-profit company	84	28.5 %	78	92.9 %
Not-for-profit company	63	21.4 %	61	96.8 %
Service Setting				
Hospital only (in-patient/out-patient)	218	73.9 %	199	91.3 %
Community only	13	4.4 %	13	100 %
Both hospital and community	64	21.7 %	63	98.4 %
Total	295	100 %	275	93.2 %

* Significant difference in response rates between responders and non-responders

Most organisations offered a range of cancer services (Table 2), with 93.9% providing specialised medical services and 76.0% providing various combinations of supportive care and survivorship services for in-patients and/or outpatients. Significant differences were observed between the ownership of a cancer service and the types of services and settings of the services (Table 2). With and without statistically adjusting for remoteness, for-profit companies were less likely than government operated services to provide chemotherapy (*p* < 0.001), cancer surgery (*p* < 0.001), palliative care (*p* < 0.001) and survivorship services (*p* < 0.001). They were also less likely to own a cancer services that provided care in community settings (*p* = 0.004) or to cancer survivors in their place of residence (*p* < 0.001). Conversely, for-profit companies were more likely to own a day hospital where no inpatient care for overnight stay was available (*p* < 0.001). However, after adjusting for remoteness, for-profit companies were significantly less likely than government operated services to own cancer services that provided both in-patient and out-patient care (unadjusted OR 0.28 (95% CI: 0.13–0.42) *p* < 0.001). Similarly, not for-profit companies were significantly less likely than government operated cancer services to provide chemotherapy (*p* = 0.001), palliative care (*p* = 0.001) and services to cancer survivors in their place of residence (*p* = 0.001). In contrast to for-profit companies, not for-profit companies were less likely to provide radiotherapy (*p* = 0.001).

**Table 2:** Ownership of cancer services and service settings

	Government operated		For-profit company		Not-for-profit company		Total	
	n	%	n	%	n	%	n	%
	Reference category		Relative risk	(RR 95% CI)	Relative risk	(RR 95% CI)		(Rate 95% CI)
Chemotherapy	127	93.4%	41	52.6%	45	73.8%	213	77.5%
			RR 0.57 ***	(0.32–0.77)	0.80 **	(0.57–0.94)		(72.2–82.0%)
Cancer surgery	86	63.2%	19	24.4%	37	60.7%	143	52.0%
			RR 0.31 ***	(0.17–0.50)	0.86	(0.60–1.11)		(46.1–57.8%)
Radiotherapy	45	33.1%	39	50.0%	8	13.1%	92	33.5%
			RR 1.38	(0.95–1.82)	0.32 **	(0.14–0.66)		(28.1–39.2%)
Palliative care	118	86.8%	14	17.9%	41	67.2%	173	62.9%
			RR 0.19 ***	(0.07–0.33)	0.75 **	(0.53–0.92)		(57.1–68.4%)
Survivorship/supportive care (total)	119	87.5%	34	43.6%	54	88.5%	209	76.0%
			RR 0.47 ***	(0.30–0.67)	0.99	(0.81–1.08)		(70.6–80.7)
Allied health	119	87.5%	31	39.7%	51	83.6%	201	73.09%
			RR 0.34 ***	(0.20–0.57)	0.91	(0.70–1.03)		(67.6–78.0)
Wellness services	34	25.0%	6	7.7%	32	52.5%	72	26.2%
			RR 0.25 **	(0.10–0.58)	1.92 **	(1.30–2.56)		(21.3–31.7)
Complementary medicine	27	19.9%	11	14.1%	33	54.1%	71	25.8%
			1.62	(0.89–2.57)	0.29 ***	(0.16–0.55)		(21.0–31.3)
Survivorship clinic	30	22.1%	9	11.5%	18	29.5%	57	20.7%
			RR 0.40 *	(0.17–0.83)	1.08	(0.59–1.77)		(16.4–25.9)
Hospital in & out-patient	94	69.1%	27	34.6%	43	70.5%	164	59.6%
			*RR 0.88*	(0.38–1.25)	0.70	(0.29–1.14)		(53.7–65.3)
Hospital in-patient only	4	2.9%	2	2.6%	5	8.2%	11	4.0%
			RR 1.04	(0.18–5.97)	3.35	(0.90–10.42)		(2.2–7.0)
Day hospital/out-patient only	34	25.0%	45	57.7%	8	13.1%	87	31.6%
			RR 2.56 ***	(2.78–10.13)	0.62	(0.28–1.20)		(26.4–37.4)
Community Clinic/Centre	49	36.0%	12	15.4%	15	24.6%	76	27.6%
			RR 0.46 **	(0.17–0.72)	0.74	(0.42–1.16)		(22.7–33.2)
Home/Residential Care Visits	63	46.3%	4	5.1%	9	14.8%	76	27.6%
			RR 0.11 ***	(0.02–0.18)	0.32 ***	(0.16–0.59)		(22.7–33.2)
Total	136	100%	78	100%	61	100%	275	100%

Association between cancer service or service setting and ownership after adjusting for remoteness (major cities, rural, remote)

*p < 0.05; **p < 0.01; ***p < 0.001

Data about the range of cancer services and settings were then combined to create a composite score designed to reflect the overall comprehensiveness of cancer services (Table 3). In keeping with the previous findings in Table 2, both before and after adjusting for remoteness, government operated services were more likely to provide comprehensive cancer care (*p* < 0.001), and for-profit companies were most likely to own a service that provided only one or two types of cancer services and in a limited range of settings (*p* < 0.001). Although the comprehensiveness of service provision varied significantly according to remoteness (all tests *p* < 0.001), no linear association with remoteness was observed (PLUM ordinal regression test that parallel lines are the same *p* = 0.002).

**Table 3:** Comprehensiveness of cancer service provision according to ownership

Range of Services & Settings ^b^	Government operated ^a^		For-profit company		Not-for-profit company		Total	
			n	%	n	%		
			Odds Ratio	(OR 95% CI)	Odds Ratio	(OR 95% CI)		
Limited range (score 1 or 2)	19	14.0%	52	66.7%	16	26.2%	87	31.6%
			OR 6.88 ***	(3.27–14.47)	OR 1.4	(0.62–3.17)		
Moderate range ^a^ (score 3 to 5)	60	44.1%	24	30.8%	36	59.0%	120	43.6%
Broad range (score 6 or 7)	57	41.9%	2	2.6%	9	14.8%	68	24.7%
			OR 0.05***	(0.01–0.25)	OR 0.16***	(0.07–0.40)		
Total	136	100%	78	100%	61	100%	275	100%

***p < 0.001; association between cancer service or service setting and ownership after adjusting for remoteness (major cities, rural, remote) [19]

^a^ reference categories; ^b^ Score calculated from types of services and settings with one point each for: for chemotherapy, surgery, radiotherapy, palliative care, supportive care, both inpatient and outpatient (hospital and/or community) settings, and provision of home/residential care visits

### Service gaps

Participants were asked an open-ended question about the most important service gap in their region. In case there were other important service gaps that were less pressing, the first question was followed by a second open-ended question about any other important needs. Most, 73.1% (*n* = 201) answered the first question and 15.6% (*n* = 43) also answered the second. Significantly lower response rates to the first question were observed for those in administrative roles only (OR 0.39, 95%CI 0.20–0.76, *p* = 0.005, reference category: HCP and administrative) and from participants located in major cities (OR 0.30, 95%CI: 0.15–0.57, *p* < 0.001, reference category: remote). No differences in response rates were observed according to the ownership of the organisation nor cancer service setting. There were no observed differences in the characteristics of the responders who answered the second question compared with those who only answered the first.

The open-ended responses to the first question were coded into four major categories and weighted according to the number of respondents from each region (i.e. Primary Health Network) (Table 4). Survivorship and supportive care services included services provided by allied health or complementary medicine practitioners, and psychosocial, survivorship, rehabilitation, and wellness services. Specialist oncology services included oncologists, chemotherapy, radiotherapy and cancer surgery. Palliative care services included palliative care physicians, palliative in/out-patient beds, palliative home care and hospices. General cancer service resources included the need for more nursing staff, inpatient beds or staff support, along with resources to support better integrated care, including the need for cancer care coordinators. Analysis of the 43 responses to the second question demonstrated that the need for specialist oncology or palliative care services had been prioritised over the other two categories. Only three respondents (all located in major cities) stated there were no important service gaps in their region.

**Table 4:** Most important cancer service gaps

	Major Cities ^a^		Regional		Remote		Total	
	n	%	n	%	n	%	n	%
			OR	(95% CI)	OR	(95% CI)		(95% CI)
Survivorship/Supportive Care ^a^	41	61.2%	30	50.0%	29	39.2%	100	49.6%
Weighted count ^b^	33	56.9%	33	51.6%	30	38.7%	95	47.9%
								(43.6–57.4%)
Specialist Oncology Services	4	6.0%	9	15.0%	21	28.4%	33	16.4%
Weighted count ^b^	2	3.4%	7	10.9%	22	28.8%	32	16.1%
			2.67	(0.74–9.65)	6.16**	(1.87–20.23)		(11.9–22.2%)
Palliative Care/Hospice	9	13.4%	15	25.0%	9	12.2%	33	16.4%
Weighted count ^b^	9	15.5%	15	23.4%	10	13.0%	33	16.7%
			2.18	(0.82–5.09)	1.40	(0.48–4.07)		(11.9–22.2%)
General Cance Service Resources	10	14.9%	6	10.0%	15	20.3%	32	15.9%
Weighted count ^b^	14	24.1%	9	14.1%	15	19.5%	38	19.4%
			0.80	(0.26–2.50)	1.98	(0.76–5.20)		(11.5–21.6%)
Total	67	100%	60	100%	74	100%	201	100%

**p = 0.003; ^a^ reference categories for multinomial logistic regression of service gaps (excluding none) and remoteness (major cities, rural, remote), [19] after adjusting for respondent’s role and cancer service ownership; ^b^ count weighted by number of respondents per Primary Health Network region: missing responses *n* = 77

Substantially more providers identified survivorship and supportive care services as the most important service gap in their region (standardised rate: 47.9, 95%CI: 43.6–57.4%) (Table 4). The proportion was significantly higher than the 19.4% (95%CI: 11.5–21.6%) of providers who identified general cancer service resources, 16.7% (95%CI: 11.9–22.2%) who identified palliative care services, and 16.1% (95%CI: 11.9–22.2%) who identified specialist oncology services as the most important service gap in their region (X_2_ Goodness-of-fit (3, *N* = 201) = 56.2, *p* < .000).

Even well-resourced services were challenged:

“*The service we provide is very comprehensive, but the difficulty in discharging elderly patients who have limited support is a significant issue.”*

After adjusting for the respondent’s role and cancer service ownership, the only significant association between service gaps and remoteness was the higher need for specialist oncology services in remote regions of Australia (OR 6.16, 95%CI: 11.87–20.23, *p* = 0.003, reference category: Survivorship cancer services). In remote regions, additional specialist oncology services that had not been included in the ‘most important service gaps’ yet were listed as an ‘other important service gaps’, were paediatric and adolescent oncology services and telehealth services.

One respondent further articulated the complex challenges of providing and coordinating interdisciplinary care for cancer survivors living in rural and remote regions:

*“Rural patients don’t do as well in cancer survivorship due to the difficulties associated with treatment access and their side effects, especially fatigue preventing them from pursuing ongoing management. Some can’t face the travel or thought of being away from home in the first instance. Cost associated with seeking lengthy treatment is also prohibitive.”*

Respondents in all regions emphasised the need for improved co-ordination of cancer services, especially for “*complex patients and for social, economic, culturally diverse communities”* and patients requiring services from multiple sites and geographical locations. This included implementing systems to improve the planning, coordination and integration of cancer care between secondary and primary care services, and between the public and private sectors.

## Discussion

This national survey was the largest and most comprehensive of its kind to have been conducted in Australia, identifying 295 healthcare organisations in the public and private health sectors and in hospital or community-based settings with dedicated cancer services in 2016. The wide range of cancer services, settings and ownership highlights the complexity of the Australian healthcare system that cancer survivors must navigate. Cancer services that aim to meet the broader bio-psycho-social needs and long-term care needs of cancer survivors were most commonly identified by providers as the most important service gap in their region. To some extent, this is a positive finding as it suggests that aside from some remote regions in Australia, generally there is adequate provision of core cancer treatment services. Nevertheless, like other high-income countries there is ‘still room for improvement’ [21].

Whilst many of the healthcare organisations surveyed offered a combination of services, a substantial proportion (31.9%), particularly in the private sector (66.7%), only provided one or two types of cancer services and in a limited range of settings. A concern with selective service provision by individual organisations is the challenge of integrating and coordinating care across the various services that cancer survivors need to access [15, 22, 23]. Indeed, the need for improved integration of services was emphasised in the qualitative comments from providers. Whilst it is possible that this pattern of service provision reflects the private sector filling specific regional service gaps in radiotherapy for example [24], privately-owned organisations were significantly less likely to provide palliative care, home care or supportive care services, despite the latter being most commonly identified by providers as the most important service gap in their region.

Another important service gap was the need for more specialist oncology services in remote regions of Australia. The challenges of providing healthcare to cancer survivors living in non-metropolitan regions of Australia and their subsequent worse health outcomes are well documented [1, 12, 24–26]. The Regional Cancer Centre Initiative, established in 2010, has focused on expanding chemotherapy and radiotherapy services into non-urban regions, engaging the private sector to fill service gaps, and developing other models of care such as regional paediatric shared care, regional outreach services, and telehealth services [26, 27]. Notwithstanding these initiatives, the findings from this survey confirm there are ongoing deficiencies with providing comprehensive cancer care in many rural and remote regions of Australia.

Irrespective of population density however, providers identified survivorship and supportive care services as the most important service gap in urban, rural and remote regions alike. This was despite the high proportion of cancer services with allied health (87.5%) – a rate slightly lower than 90% in the 2005 survey of regional and remote cancer hospitals in Australia [12]. Coupled with the finding that less than a third of the organisations surveyed provided focused services such as survivorship clinics, the results point strongly towards persisting service gaps in supportive care and survivorship services across Australia. Such findings add weight to previous research both in Australia [25, 28] and internationally [29] that consistently documents a broad range of unmet bio-psycho-social needs of cancer survivors throughout the cancer continuum trajectory [25, 28, 29].

The affordability of supportive care and survivorship services in all regions of Australia and equitable access to comprehensive cancer care for cancer survivors living in more remote areas were other concerns highlighted by providers. Although healthcare in Australia is rated as one of the best in the world, the country ranks much lower in the provision of equitable care [30, 31]. High out-of-pocket costs relative to income already adversely affect over a third of people diagnosed with cancer in Australia [11]. Expenses have been found to be disproportionately higher for cancer survivors who live outside metropolitan areas, require radiotherapy, or have private health insurance [11].

The persisting under provision of supportive care and survivorship services identified by this survey, therefore adds weight to claims that current health service planning and funding policies in Australia are yet to adequately incentivise the private sector to provide other essential, yet potentially less profitable supportive care and survivorship services [31, 32]. For example, most healthcare accessed outside of public hospitals, either as an inpatient or outpatient is funded through a fee-for-service model. However, unlike services provided by a medical practitioner, there is limited public and private insurance rebates for allied health and nursing services, and no rebates for these practitioners participate in activities such as case conferences, cancer care coordination, nor to provide home or residential care. Healthcare organisations must therefore either absorb the additional costs of providing comprehensive, multidisciplinary cancer care or pass them directly to patients [31].

Similarly, despite calls for more flexible funding arrangements for palliative care [32], few rebates are available for non-medical practitioners to provide palliative care services in community or homecare settings. Coupled with an ongoing undersupply of palliative care physicians, without radical changes to the management and funding of survivorship and palliative care services, it is difficult to see how many of the proposed indications for outpatient palliative care referrals [33] will be actioned in many parts of Australia.

Like all nations, there are ongoing concerns about the financial sustainability of the Australian healthcare system and how best to meet the ongoing unmet needs of cancer survivors [25, 28]. The landmark US Institute of Medicine (IOM) report, *From Cancer Patient to Cancer Survivor: Lost in Transition,* highlighted that cancer survivors could potentially benefit from the types of treatment programs considered to be part of ongoing cancer survivorship care [34]. Greater integration with primary care for post-treatment cancer services has been proposed as a key mechanism for providing efficient, coordinated survivorship care and improving the sustainability of national cancer services [23, 35]. However, Australia already has a strong primary care sector, yet the findings from this study suggest that substantial gaps in survivorship and supportive care services remain. Indeed, improved integration between primary and secondary care could support and encourage primary care physicians (General Practitioners) to undertake a needs assessment for their patients and assume the responsibility of surveillance for low risk patients [35]. However, focused planning and funding of allied health and nursing services will still be required to address the broader bio-psycho-social needs of cancer survivors and help coordinate survivorship care [8].

Another notable finding from the survey, was that around a quarter (26%) of the organisations in both the public and private sectors provided integrative oncology (IO) where complementary medicine (CM) services were provided to in-patients or out-patients. Whilst this rate was much higher than another recent estimate from a less representative survey [15], it remains lower than estimates from a western European survey where up to half of cancer services provide IO [36]. Australian cancer survivors are high users of CM therapies and 83% would prefer to access CM through their cancer services in an IO setting [37]. Given the growing evidence-base supporting the use of a limited range of CM therapies for concomitant cancer care [38], the increasing adoption of an IO approach by Australian cancer services may be appropriate by helping to foster safer, more effective, patient-centred care in this clinical setting [39].

This study has several strengths and weaknesses. The use of a short, online survey tool was acceptable to participants as is testament to the very high survey response and completion rates of most questions. As such, the study demonstrates the feasibility of conducting national health service surveys in small to medium sized countries that include both the public and private health sectors operating in hospital and community settings. Such health services data can be used to provide important contextual information for interpreting and acting upon national/local surveillance data and global surveillance data such as the CONCORD program [3]. A disadvantage of using a short survey was the lack of detailed information collected. Nevertheless, the survey lays the groundwork for ongoing longitudinal surveys with more specific questions about the services provided and the unmet service gaps that were identified.

Other study weaknesses included the exclusion of some palliative care services, for example, stand-alone hospices that were not owned by an organisation with a dedicated cancer service were excluded. Notwithstanding, the proportion of palliative care services identified in regional and remote Australia was slightly higher (29%) compared to 24% in a 2009 survey [12], suggesting that most palliative care services were included. The views about important service gaps from respondents working in major cities and in administrative roles were also underrepresented. This was partially adjusted for using hierarchical logistic regression and rates were also standardised by number of respondents per PHN region. However, the wide confidence intervals for the odds ratios means that it is only reasonable to make claims about the direction but not the magnitude of the odds.

Finally, cancer consultation/treatment rooms owned by small operators were not sampled. Neither were the views of cancer survivors and their care-givers that would provide important first-hand information about unmet needs. Further, due to the nature of the open-ended questions, it was not possible to extract detailed information about the service gaps identified by providers. Future research is needed to quantify which specific services are missing, disjointed or fragmented and in what regions of Australia, the extent to which specific service gaps are widening or narrowing, and the extent to which cancer services are meeting the needs of patients and their caregivers. This aligns with international calls for detailed, longitudinal, mixed-method research that examines unmet cancer care needs from the perspectives of all stakeholders (providers, patients and caregivers) [29].

## Conclusion

According to the providers in this national Australian survey, the most important cancer service gaps in their region were those aimed at meeting the broader bio-psycho-social needs and long-term care needs of cancer survivors. Despite this being a positive finding, as it suggests that aside from some rural and remote regions there is adequate provision of core cancer treatment services across Australia, there is still room for improvement. Survivorship and supportive services are mostly accessed in community and home-care settings that are predominantly funded by a fee-for-service arrangement and rely heavily on out-of-pocket payments from cancer survivors. Whilst the addition of privately-operated cancer services to supplement a baseline of public health services may have helped fill some service gaps in some parts of Australia, further research and innovative changes to service delivery and funding mechanisms are required to ensure that this mixed public-private health service arrangement provides integrated and equitable cancer services to survivors throughout the continuum of their cancer care.

## Additional file

Additional file 1: Questionnaire for cancer services. Outline of questions relevant to the data reported in this paper. (DOCX 15 kb)

## Abbreviations

GP: General Practitioner
LHD: Local Health District
PHN: Primary Health Network
