# Supplementary material for: Coverage of cancer services in Australia and providers’ views on service gaps: findings from a national cross-sectional survey
Source: BMC Cancer. 2019 Jun 11;19:570. doi: 10.1186/s12885-019-5649-6 (PMC6560726; doi:10.1186/s12885-019-5649-6)
Supplement: Supplementary file 1 — Questionnaire for cancer services. Outline of questions relevant to the data reported in this paper. (DOCX 15 kb) [file 12885_2019_5649_MOESM1_ESM.docx]

1. General information:

Organisation or hospital name: __________________________________________________________________________

Cancer service name (if different to above): __________________________________________________________________________

___________________________________________________________________________

Post code: __________________________________________________________________

Email address: ______________________________________________________________

2. Which would accurately describe your position in the organisation? (select ALL that apply)

I am in administration / management

I am a health care professional

Other (please specify) ______________________________________________________

The following section describes the provision of oncology services

3. What type of organisation owns the oncology service? Please select one only.

- Government
- Limited Company
- Not-for-profit organisation
- Registered charity
- Small business
- Don’t know
- Other please specify ________________________________________________

4. Which of the following oncology services are provided (please select all that apply):

- Chemotherapy
- Radiotherapy
- Surgery
- Survivorship clinics
- Supportive care and allied health
- Palliative oncology care
- Wellness
- Other please specify ___________________________________

5. Where are these cancer services provided? (select ALL that apply)

- Hospital setting: in-patient beds
- Hospital setting: out-patient clinic or other room
- Community setting: clinic or centre
- Home visits / Residential Care visits
- Other please specify ___________________________________________________

6. Please list any important service gaps in cancer care in your district / region

Most important unmet need ___________________________________________________

Important unmet needs _______________________________________________________

Comments__________________________________________________________________

This next section focuses on Complementary Medicine Services offered through the oncology service

The following definitions are provided for your information

Complementary Medicine – any traditional or natural therapy/practice e.g. oncology massage, acupuncture, naturopathy, chiropractic, osteopathy, therapeutic touch, reiki, aromatherapy, meditation, relaxation, yoga, tai chi, music or art therapy

Integrative Medicine – practitioners who combine evidence-based conventional Western Medicine with

7. Does your cancer service offer any Complementary Medicine or Integrative Medicine services? (select ONE only)

- Yes (go to Q8)
- No (go to Q41)
- No, not anymore (go to Q41)
- No, we never have (go to Q41)
- Don’t know (go to Q41)
- Other (e.g. planning to provide) Please specify then (got to Q41)_________________

We would like to ask you some questions about the different types of Complementary Medicine and/or Integrative Medicine your service provides.
